# Sulpho-Carbolic Acid—A Valuable Drug

**Published:** 1908-06-15

**Authors:** Elgin Ma Whinney

**Affiliations:** Chicago


### SULPHO-CARBOLIC ACID—A VALUABLE DRUG.

By Elgin Ma Whinney, D. D. S., Chicago.

Several years ago I called attention to the usefulness of pheno-sulphonic acid in the treatment of large pus pockets in the alveolar process about the roots of teeth. The value of this preparation has never gained much recognition, principally because of the difficulty of getting the pure drug. I think Dr. George W. Cook was first to suggest making it by combining equal parts of melted phenol crystals and C. P. sulphonic acid, heating slightly to facilitate union. A good deal of difficulty has been experienced in getting the necessary pure drugs with which to make the combination and without which union will not take place. About two years ago I learned that Merck makes this preparation and supplies it to the trade under the name of sulpho-carbolic acid. After two years' trial I find Merck's preparation even more useful than that I formerly employed. I recommend it for the treatment of large alveolar absorptions about the apices of roots of teeth in chronic alveolar abscesses. It should be used to burn out these pockets in exactly the same manner as most dentists have been using phenol—the root canals should first be cleansed and dentine deodorized. The pocket and sinus should next be freely washed with a saturated solution of soda bicarbonate to cleanse and empty them of pus, when after the necessary precautions are taken to avoid burning the soft tissues the sulpho-carbolic acid is forced through the root canal and out the sinus. In case of recent origin or semi-acute cases where there is no perceptible bone absorption, this agent is not indicated. I heartily recommend it in the treatment of caries of bone and necrosis about the jaws in exactly the same manner as we now use aromatic sulphuric acid.

I have previously suggested its value in treatment of deep pus pockets in pyorrhea alveolaris both before and after scaling. I now wish to recommend its application to the necks of teeth which are covered with hard black serumal calculus. All who have had experience know that this variety of deposit is very hard and often exceedingly difficult to remove, especially when high up under the gum tissue and more especially when deposited between the roots of upper molars. It is now my practice to treat these cases with sulpho-carbolic acid, full strength, three or four times before scaling is attempted. A platino-iridium broach wrapped with a few strands of cotton should be used, with which to apply it. The teeth necks should be first cleansed of debris and mucus so the medicine can penetrate the calculus. It is best to take two or three teeth at a time and keep them dry during, and for a minute after applying. After which they should be flooded with a warm antiseptic solution. No special precautions are necessary so far as the soft tissue is concerned as it is not very escharotic, in fact, I allow it to flow around the gum margins freely and find it relieves the hyper-sensitiveness and inflammation of gums which is usually present in these cases. I repeat this application three or four times two days apart. I find by experience that the calculus is much easier removed after these applications; indeed, in many cases the patient will have removed much of it by the use of the tooth brush during the days between applications. In many instances I have brought away large pieces of calculus from under the gums on the cotton wrapped broach while applying the medicine.

A case in point will illustrate the benefit of this treatment. About two years ago a gentleman came by appointment to have his teeth cleaned. Upon examination I found the gum tissue and alveolar border around every tooth had been absorbed and ulcerated away until more than half the length of the teeth roots stood exposed. They were, in fact, not exposed for they were covered by a thick coating of black or brown calculus. A sitting of two hours was required to scale one tooth, so hard was this deposit. When I realized that I had about thirty such teeth to scale, I became discouraged and so did the patient. I concluded to try the sulpho-carbolic treatment. After five applications I scaled and polished the remaining teeth in a single hour. I could relate similar experiences with probably two hundred cases until at the present time it has become my regular method of treatment in all bad serumal calculus cases.

In very deep pyorrhea pockets where the gum is nearly in normal position but sensitive and highly inflamed and the deposit is consequently very difficult of access I simplify the case by packing the pocket with a rope of cotton or gauze with a 25 per cent aqueous solution of sulpho-carbolic acid. By allowing the pack to remain for twenty-four hours, I not only soften the deposit, but benefit the necrotic area, as well as open the margin of the pocket so I can get free access and permit of scaling and polishing without lacerating the gum margin, or producing much pain. I find this drug penetrates inflamed gum tissue and consequently its germicidal power is of great value in these pus cases.

This question naturally arises: "What effect does the application of this agent have upon the cementum?" In answer to which I can only say I have never been able to see any bad effects. I do not think the drug remains in contact with the cementum long enough to decalcify it in the least.

I also make use of this drug to aid in removal of pulp nodules and to open up small canals which are closed by dried blood or hardened pulp tissue. I find it does quite as well as sulphuric acid and does not irritate the tissues in the apical space nearly so badly if a little should accidently pass through the apical foramen as does the latter preparation.—*Northwestern Dental Journal.*

